# Efficacy and Safety of Primary Glaucoma Device Implantation Surgery in Exfoliative Glaucoma: A Retrospective Consecutive Case Series

**DOI:** 10.1155/2020/3168253

**Published:** 2020-07-21

**Authors:** Sang Yoong Park, Sang Wook Jin

**Affiliations:** ^1^Dong-A University, College of Medicine, Busan, Republic of Korea; ^2^Department of Ophthalmology, Dong-A University, College of Medicine, Busan, Republic of Korea

## Abstract

**Purpose:**

To evaluate the efficacy and safety of primary glaucoma drainage implant (GDI) surgery for exfoliation glaucoma (XFG).

**Methods:**

This study was a retrospective, consecutive case series study including 36 eyes of 36 patients with XFG who underwent primary GDI surgery. Intraocular pressure (IOP), the mean deviation (MD) from the visual field exam, corneal endothelial cell density (ECD), and the number of topical antiglaucoma agents used during the preoperative and postoperative periods were retrospectively analyzed. Surgical success was defined by the following criteria: (1) IOP ≤ 18 mmHg and an IOP reduction of 20% with 1 or no medication; (2) IOP ≤ 15 mmHg and an IOP reduction of 25% with 1 or no medication; and (3) IOP ≤ 12 mmHg and an IOP reduction of 30% with 1 or no medication. The probability of success of GDI surgery was determined via Kaplan–Meier survival analysis.

**Results:**

The preoperative IOP was 25.9 ± 4.7 mmHg, and the postoperative IOP at 24 months was decreased to 14.2 ± 3.6 mmHg (*p* value < 0.001). The postoperative MD and ECD were similar to baseline (MD *p* value = 0.155; ECD *p* value = 0.055). However, a significant reduction in the number of antiglaucoma agents was observed (*p* value < 0.001). The surgical success rates were 77.8%, 63.9%, and 55.6% at 24 months for criteria 1, 2, and 3, respectively. Early hypotony (4 patients, 11.1%) and persistent corneal edema (5 patients, 13.9%) were the most common early and late postoperative complications, respectively.

**Conclusions:**

In XFG, primary GDI surgery reduced IOP by 45.2% and had a 77.8% success rate according to criteria 1 at 24 months postoperatively. However, considering that ECD reduction continues to decline over time, primary GDI surgery should be carefully considered in XFG.

## 1. Introduction

Exfoliative glaucoma (XFG) is the most common identifiable cause of open-angle glaucoma and develops as a result of exfoliation syndrome (XFS), a generally progressive age-related systemic disorder of the extracellular matrix in which white fibrillogranular material is deposited in ocular tissues, including the anterior lens surface, trabecular meshwork, and zonule; XFG is considered more severe than primary open-angle glaucoma (POAG) [1–3].

In general, XFG has a more rapidly progressive course than POAG and is associated with higher mean intraocular pressure (IOP), higher peak IOP, wider IOP fluctuation, and greater optic disc damage and glaucomatous visual field defects [3–6]. Moreover, XFG is more resistant than POAG to medical therapy and has a higher medical treatment failure rate. Therefore, when XFG patients need greater IOP reduction or a lower target IOP, surgical intervention should be considered the treatment of choice [6–9].

Surgical intervention for XFG has traditionally included trabeculectomy and glaucoma drainage implant (GDI) surgery. Trabeculectomy currently remains the standard surgical procedure for XFG that is not controlled by medical therapy or laser therapy [10, 11]. Previous studies have suggested that trabeculectomy has good efficacy and achieves safe outcomes in XFG [12, 13]. Nevertheless, because of the higher failure rate and early postoperative complications of trabeculectomy, there has been a shift towards GDI surgery as the primary choice in XFG [14, 15]. The Tube Versus Trabeculectomy (TVT) study showed that tube shunt surgery had better efficacy and safety than trabeculectomy. However, the TVT study included only 4 XFG patients (4%) [16]. To the best of our knowledge, the studies to date that have evaluated the response of XFG to GDI surgery are insufficient.

The purpose of this study was to evaluate the efficacy and safety of primary GDI surgery in XFG patients whose IOP is uncontrolled despite the maximum tolerated medical and laser therapy.

## 2. Methods

This study was a retrospective, consecutive case series study including 36 eyes of 36 patients with XFG who underwent primary GDI surgery between January 2014 and December 2019 at the Dong-A University Medical Center (Busan, Republic of Korea). This study was approved by the Institutional Review Board of Dong-A University. Informed consent was obtained from each participant, and every individual on the study team adhered to the tenets of the Declaration of Helsinki.

XFG was diagnosed as glaucomatous optic neuropathy accompanied by exfoliation material deposition on the anterior lens capsule and/or pupillary margin.

Eligible patients met the following criteria: (1) age: 18–75 years; (2) XFG treated with primary GDI surgery because of uncontrolled IOP despite the maximum tolerated medical and laser therapy; and (3) follow-up period >24 months.

The exclusion criteria were as follows: (1) eyes with other visually significant ocular pathology (e.g., visually significant cataracts, diabetic retinopathy, retinal vessel occlusions, or macular degeneration); (2) patients on medications (e.g., steroids and hydroxychloroquine) that could affect visual sensitivity and IOP; and (3) a history of ocular surgery, including cataract operations.

If both eyes met the eligibility criteria, the right eye was selected for analysis. In monocular cases, the affected eye was used for analysis.

The patient demographics, IOP, mean deviation (MD) value from the visual field exam, central corneal thickness (CCT), corneal endothelial cell density (ECD), and number of topical antiglaucoma agents were retrospectively analyzed.

IOP was measured with a Goldmann applanation tonometer (GAT), and preoperative IOP was defined as the average of 3 consecutive measured values before GDI surgery. A visual field test was performed using the automated perimetry test (Humphrey Field Analyzer, C24-2 Swedish Interactive Thresholding Algorithm (SITA) standard program, Carl-Zeiss Meditec). CCT was measured by tomography (Pentacam, Oculus, Wetzlar, Germany), and ECD was measured by specular microscopy (Konan NSP-9900, Konan Medical, Inc., Hyogo, Japan). Complications, including hypotony, hyphema, bleb infection, shallow anterior chamber, choroidal detachment, and endophthalmitis, were analyzed.

Three levels of surgical success were defined by the criteria utilized in a previous study [17]: (1) IOP ≤ 18 mmHg and an IOP reduction of 20% with 1 or no medication; (2) IOP ≤ 15 mmHg and an IOP reduction of 25% with 1 or no medication; and (3) IOP ≤ 12 mmHg and an IOP reduction of 30% with 1 or no medication.

All surgical procedures were performed by a single glaucoma surgeon (SWJ). A fornix-based conjunctiva and Tenon's flap were prepared to place an Ahmed glaucoma valve model FP7 (New World Medical, Rancho Cucamonga, CA, USA) in the superotemporal quadrant between the lateral and superior rectus muscles. Tube priming was performed with balanced salt solution irrigation. After the plate was fixed at the sclera with 8-0 Prolene® sutures, a 5 × 5 mm limbal-based scleral flap was made to cover the silicone tube. Then, the tube was sized for placement 2 mm anterior to the limbus. A scleral track was made under the scleral flap by inserting a 23-gauge needle into the anterior chamber. The tube tip was positioned with the bevel up at a maximal distance from the corneal endothelium and anterior to the iris through the scleral track. The tube was then covered with a scleral flap and sutured with 8-0 Vicryl®. The conjunctiva and Tenon's capsule were closed with a running suture with 8-0 Vicryl®.

After the operation, topical antibiotics and anti-inflammatory agents were used for 1 month.

Statistical analyses were performed using the software program SPSS (version 20.0, SPSS Inc., Chicago, IL, USA). The probability of successful GDI surgery was determined via Kaplan–Meier survival analysis. Student's paired *t*-test or the Mann–Whitney *U*-test was used for the analysis of continuous variables. *p* values less than 0.05 indicated statistical significance.

## 3. Results

The mean age was 65.8 ± 7.4 years. Twenty-five patients (69.4%) were males. The mean preoperative IOP was 25.9 ± 4.7 mmHg. The mean preoperative MD, CCT, preoperative ECD, and number of preoperative antiglaucoma agents used were −23.6 ± 5.3 dB, 541.2 ± 25.4 *μ*m, 2149 ± 479 cell/mm^2^, and 3.9 ± 0.6, respectively (Table 1).

**Figure 1:**
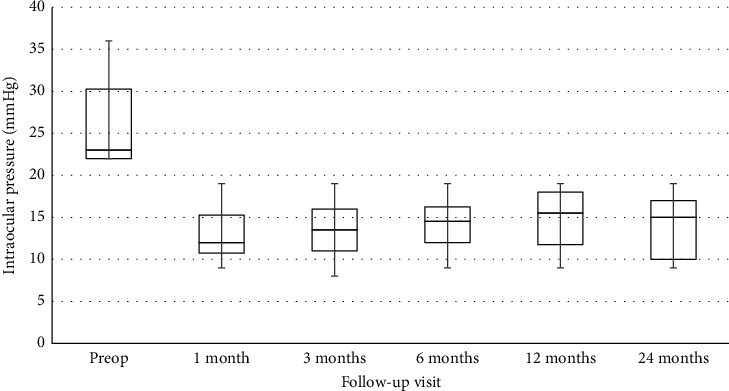
Box-and-whisker plot of the mean intraocular pressure before and after surgery. The horizontal line within each box denotes the median value; the boxes extend from the 25^th^ to the 75^th^ percentiles of each group's distribution of values; the vertical lines denote adjacent values (i.e., the most extreme values within the 1.5 interquartile range of the 25^th^ and 75^th^ percentiles of each group).

The preoperative and postoperative IOP results at each follow-up visit are shown in Figure 1. The mean preoperative IOP was 25.9 ± 4.7 mmHg, and the mean postoperative IOP at 24 months was 14.2 ± 3.6 mmHg. There was a significant reduction in IOP after GDI surgery at each follow-up visit (*p* value < 0.001).

The results obtained for the preoperative and postoperative MDs, mean ECDs, and mean numbers of antiglaucoma agents at each follow-up visit are shown in Table 2. The mean preoperative MD and ECD were −23.6 ± 5.3 dB and 2149 ± 479 cell/mm^2^, respectively. The mean MD and ECD at 24 months postoperatively were −24.5 ± 4.9 dB and 2084 ± 465 cells/mm^2^, respectively. Compared with preoperative values, these postoperative results were not significantly different (MD *p* value = 0.155 and ECD *p* value = 0.055). However, there was a significant reduction in the number of antiglaucoma agents used after GDI surgery for XFG (*p* value < 0.001).

**Figure 2:**
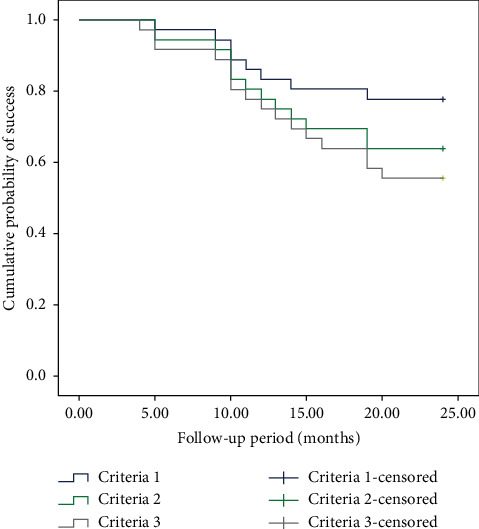
Kaplan–Meier survival analysis. The graph shows the success rates of primary glaucoma device implantation surgery according to criteria 1 (IOP ≤ 18 mmHg and an IOP reduction of 20% with 1 or no medication), 2 (IOP ≤ 15 mmHg and an IOP reduction of 25% with 1 or no medication) and 3 (IOP ≤ 12 mmHg and an IOP reduction of 30% with 1 or no medication) in XFG.

Figure 2 shows the results as the percentage of eyes with surgical success in the Kaplan–Meier survival analysis according to the three levels of surgical success. For criteria 1, the success rates of GDI surgery for XFG were 97.2% at 6 months, 83.3% at 12 months, and 77.8% at 24 months. For criteria 2, the success rates were 94.4%, 77.8%, and 63.9%, respectively. For criteria 3, the rates were 91.7%, 75.0%, and 55.6%, respectively.

Postoperative complications are presented in Tables 3 and 4. The total number of patients with early postoperative complications was 8 22.2%. Early hypotony (4 patients, 11.1%) occurred with the highest frequency, and additional complications were as follows: choroidal effusion (4 patients, 11.1%), hyphema (2 patients, 5.6%), and wound leak (1 patient, 2.8%). Cystoid macular edema and endophthalmitis did not occur in this study (Table 4). The total number of patients with late postoperative complications was 11 (30.5%). Persistent corneal edema (5 patients, 13.9%) occurred with the highest frequency, and additional complications were as follows: cystoid macular edema (3 patients, 8.3%), tube erosion (3 patients, 8.3%), and choroidal effusion (2 patients, 5.5%). Persistent diplopia, endophthalmitis, and blebitis did not occur in this study.

## 4. Discussion

Trabeculectomy is generally used as the primary surgery for glaucoma. However, specific patients, such as those who have uveitic glaucoma or neovascular glaucoma or who have previously undergone conjunctival incisional surgery, are at a greater risk of surgical failure [16, 18]. The adjunctive use of antifibrotic agents, such as mitomycin-C (MMC) and 5-fluorouracil (5-FU), improves the success rate of trabeculectomy but increases the risk of bleb-related complications [19]. The increased risk of bleb-related complications has contributed to the growing use of GDI as an alternative to trabeculectomy [20]. Many studies have reported the efficacy of primary GDI surgery in different types of glaucoma. However, few studies have evaluated the efficacy of primary GDI surgery in XFG. Therefore, we evaluated the efficacy and safety of primary GDI surgery in XFG.

Previous studies have demonstrated the efficacy and safety of primary GDI surgery in XFG [16, 21]. Gedde SJ et al. demonstrated in the TVT study that the success rate was higher for tube shunt surgery than trabeculectomy based on MMC during 5 years of follow-up. Both procedures were associated with similar IOP reductions and the use of supplemental medical therapy at 5 years [16]. However, the TVT study included only 4 XFG patients (4%). Therefore, this result does not represent the efficacy of primary GDI in XFG.

We conducted our study in 36 XFG patients who underwent primary GDI surgery and found that the mean postoperative IOP at 1 month after primary GDI surgery was 13.1 ± 3.1 and reduced IOP by 49.4% from preoperative IOP in XFG. A nearly 40% IOP reduction rate lasted for 24 months. The mean postoperative IOP at 24 months was 14.2 ± 3.6 mmHg, and IOP was 45.2% lower than the preoperative value in these XFG patients. In addition, the number of antiglaucoma agents was reduced to 1.1 ± 0.6 from 3.9 ± 0.6. These reductions in IOP and the number of antiglaucoma agents demonstrate that primary GDI surgery was associated with satisfactory IOP and antiglaucoma agent management in XFG.

Previous studies on the surgical success rate of trabeculectomy in XFG have demonstrated that this approach has a surgical success rate of approximately 70–80% [22, 23]. Ehrnrooth et al. reported a 70% surgical success rate at 24 months postoperatively for trabeculectomy without antifibrotic agents. Those investigators defined surgical success as follows: (1) complete success was defined as IOP ≤ 21 mmHg achieved without additional therapy, and (2) qualified success was defined as IOP ≤ 21 mmHg achieved with a single topical medication [23]. Lim and Cha reported an 81% surgical success rate at 36 months postoperatively for trabeculectomy with MMC based on their definition of surgical success (IOP < 18 mmHg and IOP reduction ≥20% with or without medication). In our study, the success rate of primary GDI surgery for XFG according to criteria 1 was 77.8% at 24 months postoperatively. Despite differences in study conditions, such as the definition of surgical success and the use of antifibrotic agents, the success rates were similar between primary GDI surgery and trabeculectomy performed with or without antifibrotic agents in XFG. For further evaluation of the long-term efficacy after 24 months and to compare primary GDI surgery and trabeculectomy under the same conditions, a randomized comparative clinical trial of primary GDI surgery versus primary trabeculectomy for XFG is needed. In addition, we evaluated surgical success under more stringent criteria (criteria 2 or 3). XFG has a more aggressive clinical course than POAG. Therefore, a target IOP in the mid- or low-teens is needed in XFG to prevent glaucoma progression. Fontana H et al. reported 62% and 45% surgical success rates for trabeculectomy with MMC at 36 months postoperatively based on two different definitions of surgical success (IOP ≤ 15 mmHg plus an IOP reduction of 25% and IOP ≤ 12 mmHg plus an IOP reduction of 30%) [17]. In our study, the surgical success rates according to criteria 2 and 3 at 24 months postoperatively were 63.9% and 55.6%, respectively. The surgical success rate in our study at 12 months postoperatively was comparable to that of Fontana et al.

Postoperative complications after GDI surgery have been reported to include hypotony, choroidal effusion, implant exposure or migration, and corneal edema [21, 24]. Gedde et al. reported that the early and late postoperative complication rates of this procedure were 21% and 34%, respectively. The three most common early complications were choroidal effusion (14%), shallow or flat anterior chamber (10%), and aqueous misdirection (3%). The three most common late complications were persistent corneal edema (16%), persistent diplopia (6%), and tube erosion (5%) [21]. In our study, total numbers of patients with early and late postoperative complications were 8 (22.2%) and 11 (30.5%), respectively. The most common early and late complications were choroidal effusion (4 patients, 11.1%) and persistent corneal edema (5 patients, 13.9%, respectively). The postoperative complications observed in our study were consistent with the findings of previous studies.

Several studies have demonstrated that ECD is lower in XFG patients [25]. In addition, GDI surgery can cause endothelial cell loss [26, 27]. Therefore, in XFG patients, GDI surgery patients can cause further ECD reduction and serious postoperative complications, such as corneal edema. For this reason, we evaluated the changes in ECD before and after GDI surgery. Oh et al. reported that compared to preoperative findings, the number of corneal endothelial cells was significantly lower (2278 ± 565/mm^2^ vs. 2177 ± 529/mm^2^, *p*=0.043) in the central corneal area at 19.2 months postoperatively [27]. In our study, ECD at postoperative 24 months was decreased to 2149 ± 479/mm^2^ compared with preoperative ECD (2084 ± 465/mm^2^). This result was not statistically significant (*p* value = 0.055). However, considering that ECD reduction continues to decline with age, primary GDI surgery should be carefully considered in XFG. Therefore, we believe that additional studies are needed.

This study has several limitations. First, the retrospective design involving a tertiary care setting may have introduced a selection bias. However, our study was a consecutive case series. All eligible patients identified by the authors during the study period were included; therefore, bias was minimized. Second, this study did not include a trabeculectomy group for comparison with the primary GDI surgery group. Third, we excluded patients who had undergone cataract surgery before GDI surgery or had received combined surgery (GDI and cataract surgery) to exclude the IOP-lowering effect of cataract surgery. However, a phakic state may also affect proper placement of an Ahmed valve and postoperative complications. In our study, a phakic state was not reflected in the results. Fourth, anterior chamber depth (ACD) and aging are important factors when analyzing ECD reductions; however, we did not consider ACD and aging. Additional long-term studies are needed. While little is known about the efficacy and safety of primary GDI surgery in XFG, our study provides some preliminary information on the efficacy and safety of primary GDI surgery for XFG during the first 24 months after the procedure.

In conclusion, primary GDI surgery had a 45.2% IOP reduction rate and a 77.8% surgical success rate according to criteria 1 (IOP ≤ 18 mmHg and an IOP reduction of 20% with 1 or no medication) at 24 months postoperatively in XFG patients. However, considering that postoperative complications, such as ECD reduction, continue to decline over time, primary GDI surgery should be carefully considered in XFG.

## Figures and Tables

**Table 1:** Clinical and demographic data of the patients.

Characteristics	Exfoliative glaucoma (*N* = 36)
Age, yrs	65.8 ± 7.4
Male, *n*(%)	25 (69.4%)
Preoperative IOP, mmHg	25.9 ± 4.7
Preoperative MD, dB	−23.6 ± 5.3
CCT, *μ*m	541.2 ± 25.4
ECD, cells/mm^2^	2149 ± 479
Preoperative topical antiglaucoma agents, *n*	3.9 ± 0.6

Values are presented as the mean ± standard deviation unless otherwise indicated. IOP = intraocular pressure; MD = mean deviation; CCT = central corneal thickness; ECD = corneal endothelial cell density.

**Table 2:** Comparison of the mean MD, ECD, and number of topical antiglaucoma agents.

	Preoperative (*p* value^*∗*^)	6 months^†^ (*p* value^*∗*^)	12 months^†^ (*p* value^*∗*^)	24 months^†^ (*p* value^*∗*^)
MD, dB	−23.6 ± 5.3 (N/A)	−24.7 ± 5.2 (0.060)	−24.1 ± 5.2 (0.405)	−24.5 ± 4.9 (0.155)

ECD, cell/mm^2^	2149 ± 479 (N/A)	2097 ± 457 (0.058)	2112 ± 416 (0.125)	2084 ± 465 (0.055)

Antiglaucoma agents	3.9 ± 0.6 (N/A)	0.7 ± 0.5 (<0.001)	0.9 ± 0.8 (<0.001)	1.1 ± 0.6 (<0.001)

Values are presented as the mean ± standard deviation unless otherwise indicated (*p* value). MD = mean deviation; ECD = corneal endothelial cell density; N/A = not applicable. ^*∗*^Paired *t*-test; statistical significance: *p* < 0.05 vs. preoperative value. ^†^Follow-up period after glaucoma device implantation.

**Table 3:** Early postoperative complications.

	Glaucoma device implantation (*N* = 36)
Early hypotony	4 (11.1%)
Choroidal effusion	4 (11.1%)
Hyphema	2 (5.6%)
Wound leak	1 (2.8%)
Endophthalmitis	0
Cystoid macular edema	0
Total number of patients with complications^*∗*^	8 (22.2%)

Values are presented as the number of patients (percentage). ^*∗*^Some patients had more than 1 complication.

**Table 4:** Late postoperative complications.

	Glaucoma device implantation (*N* = 36)
Persistent corneal edema	5 (13.9%)
Cystoid macular edema	3 (8.3%)
Tube erosion	3 (8.3%)
Choroidal effusion	2 (5.5%)
Persistent diplopia	0
Endophthalmitis	0
Blebitis	0
Total number of patients with complications^∗^	11 (30.5%)

Values are presented as the number of patients (percentage). ^*∗*^Some patients had more than 1 complication.

## Data Availability

Data are available on request. If anyone wants data, they should contact the Institutional Review Board of the Dong-A Medical Center.
